# A comparison of the acute effects of high intensity interval training and moderate intensity continuous training on working memory and emotional state in adolescent women with subthreshold depression

**DOI:** 10.3389/fpubh.2025.1505959

**Published:** 2025-02-07

**Authors:** Shiwei Yuan, Lili Lin, Libin Liu, Xinyuan Zhang, Qian Gu

**Affiliations:** ^1^Wuxi Higher Health Vocational Technology School, Wuxi, China; ^2^School of Physical Education, Shandong University, Jinan, China

**Keywords:** depression, executive function, pleasure, arousal, exercise intensity

## Abstract

**Objective:**

Various guidelines emphasize the cognitive and emotional benefits of physical activity. However, it is not yet clear what kind of exercise intensity is suitable for individuals with subthreshold depression. Therefore, this study aims to examine the effects of high intensity interval training (HIIT) and moderate intensity continuous training (MICT) on the working memory and emotional state.

**Methods:**

Forty-nine female students with subthreshold depression (mean age 16.3 ± 0.5 yrs) completed 20-min group of sit (SIT), MICT, and HIIT on separate days in a counterbalanced order. The modified n-back task was employed to evaluate working memory. Emotional state was assessed using the Feeling Scale (FS) and Felt Arousal Scale (FAS).

**Results:**

Regarding working memory, for accuracy, SIT (82.41 ± 13.79%), MICT (81.79 ± 13.19%) and HIIT (82.06 ± 11.36%) have no significant difference (all *p* > 0.05). However, for reaction time, MICT (459.86 ± 131.47 ms) is significantly faster than HIIT (491.16 ± 115.68 ms) (*p* = 0.046), and there is no significant difference between MICT and SIT (462.71 ± 120.64 ms) (*p* > 0.05). Concerning emotional state, for arousal, FAS scores significantly increased after both HIIT (2.39 ± 1.30 to 3.76 ± 1.12, *p* < 0.001) and MICT (2.55 ± 0.88 to 3.94 ± 1.08, *p* < 0.001) compared to pre-exercise, and the increase caused by them was significantly higher than that of SIT (HIIT: *p* = 0.011; MICT: *p* < 0.01). For pleasure, the increase in FS score after MICT (−0.27 ± 1.13 to 1.06 ± 1.79) was significantly higher than that of SIT (0.32 ± 1.38 to 0.58 ± 1.53) (*p* < 0.01).

**Conclusion:**

Acute MICT can enhance working memory and improve the emotional state of adolescent women with subthreshold depression, while HIIT may decrease working memory and pleasure in this particular population.

## 1 Introduction

Depression is one of the most significant contributors to the global health burden (1), affecting approximately 322 million individuals worldwide, or 4.4% of the global population (2). Adolescence is a high-risk period for developing depression, with approximately 34% of adolescents aged 10–19 at risk of clinical depression (3). Among them, adolescent females are particularly vulnerable, exhibiting higher rates of depression compared to their male counterparts (4). This gender disparity may stem from a combination of biological factors, such as hormonal changes (5), and sociocultural factors, including societal pressures (6) and peer correlates (7) during adolescence. These challenges make adolescent females more prone to emotional dysregulation and stress, emphasizing the urgent need for targeted interventions during this critical developmental stage. Subthreshold depression, which shares clinical characteristics and risk factors with major depression (8, 9), represents a transitional phase between health and clinical depression (10). This transitional nature makes subthreshold depression a crucial target for early prevention and intervention. Adolescents with subthreshold depression not only experience emotional dysregulation (11–13) but also show cognitive deficits, such as negative cognitive bias and attention bias toward negative stimuli (14, 15), which are rooted in maladaptive cognitive schemas formed through heredity and early traumatic experiences. Addressing subthreshold depression in adolescence is essential, as it offers a unique opportunity to halt its progression to clinical depression and mitigate its associated long-term impacts.

Current interventions for adolescent depression, including pharmacological and psychological approaches, face several limitations. Drug treatments often lead to adverse side effects, such as headaches, nausea (16), and an increased risk of suicidal ideation (17, 18), while psychotherapy exhibits limited efficacy in younger populations and a high recurrence rate (19, 20). In contrast, exercise interventions have emerged as a promising alternative due to their ability to improve both depressive symptoms (21) and cognitive functions (22), particularly executive functions like working memory (23). Enhancing working memory through exercise has been associated with improved emotion regulation, reduced negative emotional responses, and activation of brain regions responsible for both cognitive and emotional regulation, such as the ventrolateral and dorsolateral prefrontal cortex (24–26).

Despite extensive research on exercise interventions for depression (27–30), most studies focus on either healthy individuals or clinically diagnosed patients, leaving adolescents with subthreshold depression underrepresented. Furthermore, adolescent females, who experience disproportionately higher rates of subthreshold depression (31), remain a particularly vulnerable and understudied group. By targeting this critical transitional phase, this study provides insights into the potential of exercise interventions to prevent the progression of subthreshold depression and improve emotional and cognitive outcomes in this population.

This research also uniquely integrates exercise intensity and its cognitive-emotional effects into a targeted intervention framework. By exploring the acute effects of different exercise intensities, including high-intensity interval training (HIIT), moderate-intensity continuous training (MICT), and sedentary activity (SIT), on emotional states and working memory, the study aims to fill gaps in the literature and inform the design of effective exercise-based interventions tailored for adolescent females with subthreshold depression. These findings are expected to have practical implications for physical education curricula and adolescent mental health strategies.

## 2 Materials and methods

### 2.1 Participants

Forty-nine female students (mean age 16.3 ± 0.5 yrs.; BMI 22.8 ± 5.2 kg m^−2^), who had not committed to regular exercise in the past six months participated in the current experiment. All participants had subthreshold depression, but there was no history of clinically diagnosed neurological or psychiatric disorders among the participants. Screening for subthreshold depression was conducted using the Center for Epidemiologic Studies Depression Scale (32, 33) (CES-D) and Beck Depression Inventory (34, 35) (BDI), with CES-D ≥ 16 points and 5 ≤ BDI ≤ 14 points as the criteria. The participants were in good physical health, and written consent was obtained from all of them. Participants were recruited from local high schools via online and offline advertisements. Initial screening involved a self-reported questionnaire, and eligible participants were invited for further assessment using the CES-D and BDI scales. Only those meeting the inclusion criteria were included. All participants provided verbal consent, and their parents provided written informed consent. The study adhered to ethical guidelines outlined in the Declaration of Helsinki, and ethical clearance was obtained from the Institutional Ethics Committee of Wuxi Higher Health Vocational Technology School (2022003).

### 2.2 Experimental protocol

A randomized crossover design was employed, and participants visited the laboratory four times. The initial visit involved completing a Physical Activity Readiness Questionnaire (PAR-Q) to ensure safety for subsequent assessments. During the subsequent visits, participants wore a heart rate monitor (Polar OH1, Kempele, Finland).

**Figure 1:**
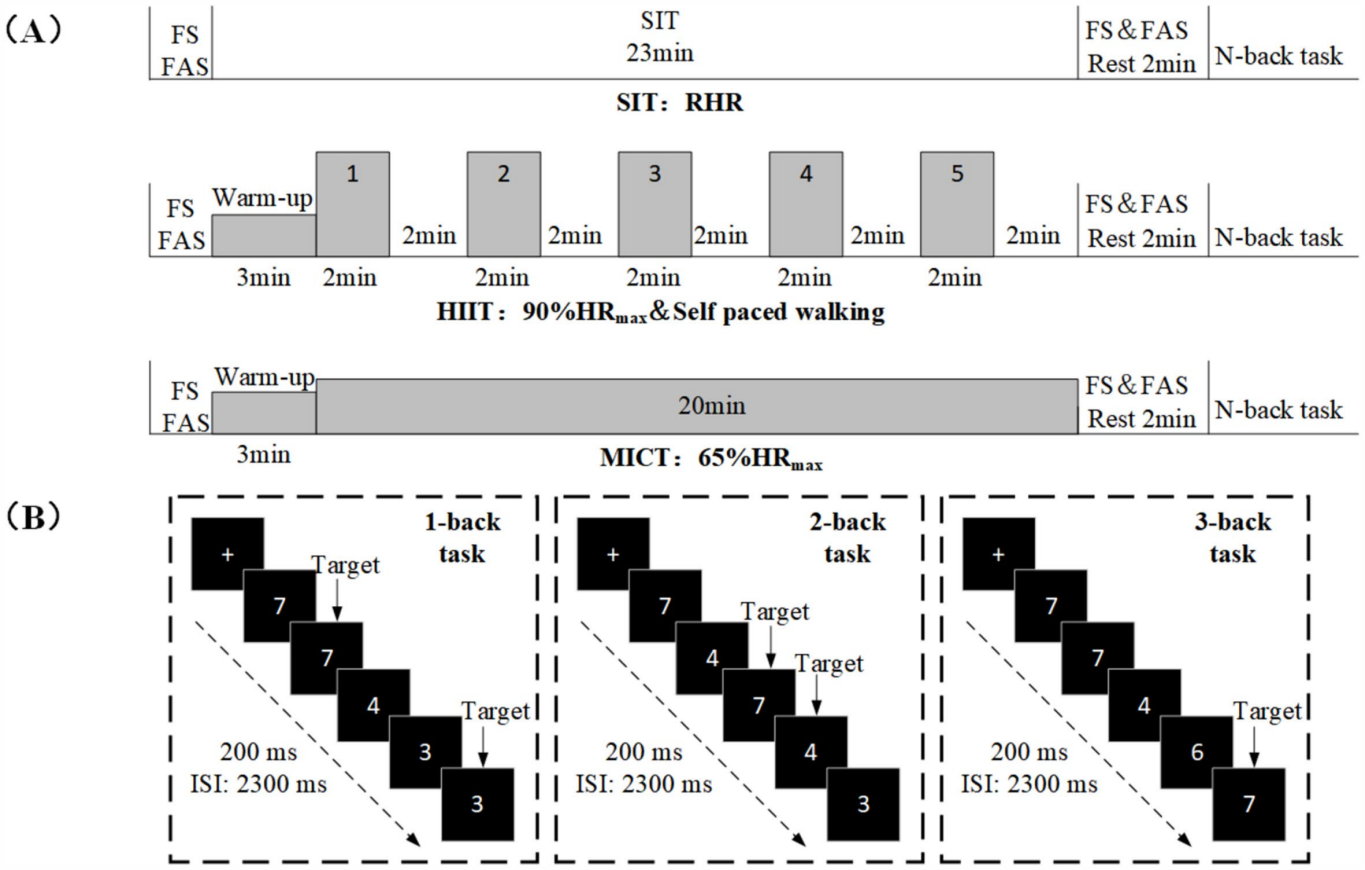
Experimental protocol. **(A)** The experimental design includes three conditions: SIT, HIIT, and MICT. **(B)** Schematic illustration of the n-back task. Examples of trials for the 1-back, 2-back and 3-back conditions of the n-back task are exemplified.

The main experiment comprised three groups: SIT, HIIT, and MICT, separated by at least one week. Participants were assigned to one of six group orders to account for experimental sequence effects through counterbalancing. The SIT protocol included 23 min sit at resting heart rate (RHR), and a 2-min cool-down. The HIIT protocol included a 3-min warm-up, 20 min of interval exercise, and a 2-min cool-down. Participants performed a warm-up and cool-down guided by a professional coach, followed by five bouts of 2-min running at 90% of maximum heart rate (HR_max_) (estimated by 220-age), interspersed with 2 min of self-paced walking. Participants in the MICT group had a 3-min warm-up, followed by 20 min of running at 65% of HR_max_, and a 2-min cool-down (Figure 1A). Two assistant testers were present during the experiment, prompting participants to adjust their pace based on heart rate (HR) responses during HIIT and MICT interventions.

Participants underwent Rating of Perceived Exertion (RPE) tests before and immediately after intervention to assess subjectively measured load intensity and experience (36). Simultaneously, the Feeling Scale (FS) and the Felt Arousal Scale (FAS) tests were conducted to evaluate emotional valence and arousal (37). Following the intervention and upon the return of heart rate to a resting level, participants underwent an n-back test to assess working memory ability. Participants were instructed to abstain from vigorous exercise and caffeine consumption for at least 24 h before each group.

### 2.3 N-back paradigm

This study utilized modified versions of the n-back task, a well-established paradigm for investigating working memory (38, 39). The task was programmed and presented using E-Prime 3.0 (Psychology Software Tools Inc., United States). The number of stimuli (ranging from 3 to 8) was displayed in the center of the screen (Figure 1B). The n-back task comprised ten blocks, totaling 136 trials, with 1-back and 2-back tasks having 4 blocks each, and the 3-back task having 2 blocks. In 1-back and 2-back tasks, each block consisted of 12 trials (four target and eight non-target trials) presented in a pseudorandomized order. The 3-back task included 20 trials (6 target and 14 non-target trials) similarly presented in a pseudorandomized order. In each trail, a single number was presented in the center of a computer screen for 200 ms, followed by an inter-stimulus interval (ISI) of 2,300 ms. The participants should respond within 1,000 ms. Responding with a left-hand press (“F” key) was required when the current stimulus matched from n-steps earlier in the sequence (i.e., target), and with the right-hand press (“J” key) when the current stimulus not matched from n-steps earlier in the sequence (i.e., non-target). Participants were required to respond as quickly and accurately as possible.

### 2.4 FS and FAS

To evaluate participants’ emotional valence and emotional activation during exercise, the FS and FAS were employed. The FS is a one-term inventory measuring the extent to which participants feel pleasant or unpleasant and ranges from “very good” (+5) to “very bad” (−5) (40). The FAS is identically a one-term inventory measuring feeling of arousal and ranges from “low arousal” (1) to “high arousal” (6) (41). Emotional valence and activation levels were collected before and immediately after the intervention.

### 2.5 Statistical analysis

The study utilized a previously obtained effect size from Kao, Westfall, Soneson, et al. (42), which was based on the impact of acute exercise intensity on executive function (*η*^2^ = 0.57). This effect size was determined in G-Power 3.1 software to estimate the necessary sample size (43). The statistical test employed was a repeated measures within-between interaction, with a significance level of *α* = 0.05 and a required power of (1 - *β*) = 0.95. Calculations showed that a minimum of 15 participants were needed to achieve the desired sample size under the current experimental design framework. In the end, 49 participants participated and completed the study.

All statistical analyses were performed using SPSS V.22 (IBM, Chicago, IL, United States). The RT and ACC of the n-back task were analyzed using a 3 (group: SIT, HIIT, and MICT) × 3 (task condition: 1-back, 2-back, and 3-back) repeated-measures analysis of variance (RM-ANOVA). Pleasure and arousal levels from the FS and FAS were analyzed using a 3 (group: SIT, HIIT, and MICT) × 2 (pre-intervention: pleasure and arousal; post-intervention: pleasure and arousal) RM-ANOVA. Partial eta squared (*η*^2^) was calculated as an effect size measure. Significance levels were set at *p* < 0.05.

## 3 Results

### 3.1 HR and RPE

**Figure 2:**
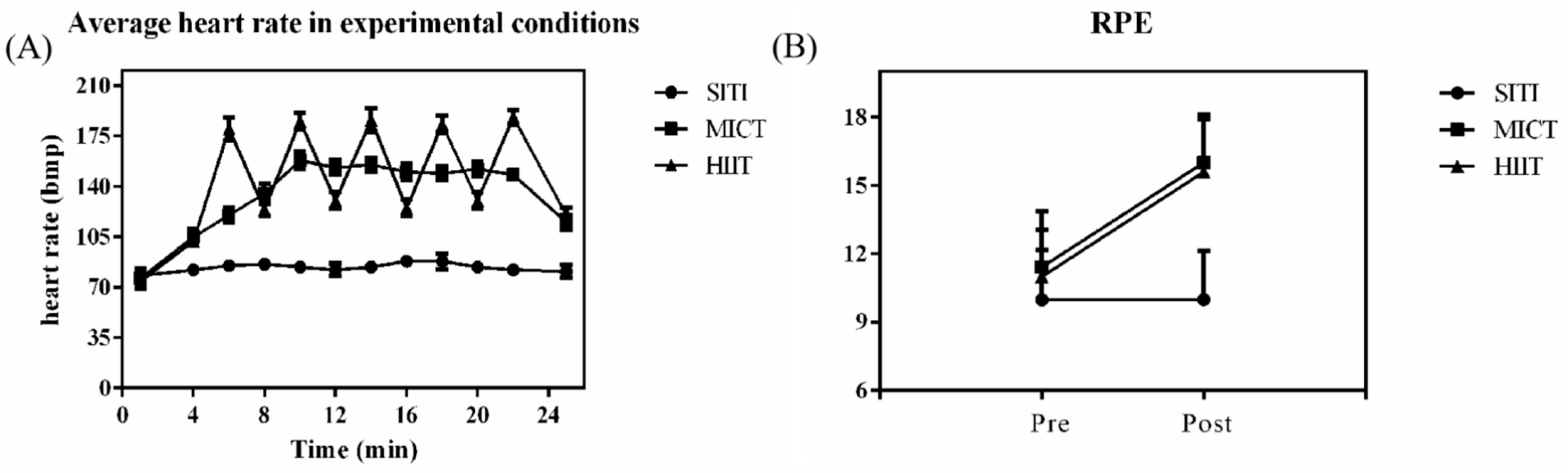
**(A)** Mean HR (± 1 SD) over the course of each experimental condition. **(B)** Average RPE in experimental condition (± 1 SD).

Physiological characteristics of different exercise intensities were explored by monitoring HR and the RPE. As depicted in Figure 2, during HIIT, the numeric range of HR and RPE were 162.26 ± 14.65 beats/min (79.15 ± 7.15% of maximum heart rate) and 14.94 ± 2.10 points, respectively. For MICT, the numeric range of HR and RPE were 138.68 ± 12.62 beats/min (67.65 ± 6.16% of maximum heart rate) and 14.65 ± 2.46 points, respectively. According to the ACSM guidelines classification (44), the overall intensity of the HIIT condition was described as vigorous and slightly above moderate, and the intensity of exercise in the MICT condition was moderate.

### 3.2 N-back results

**Figure 3:**
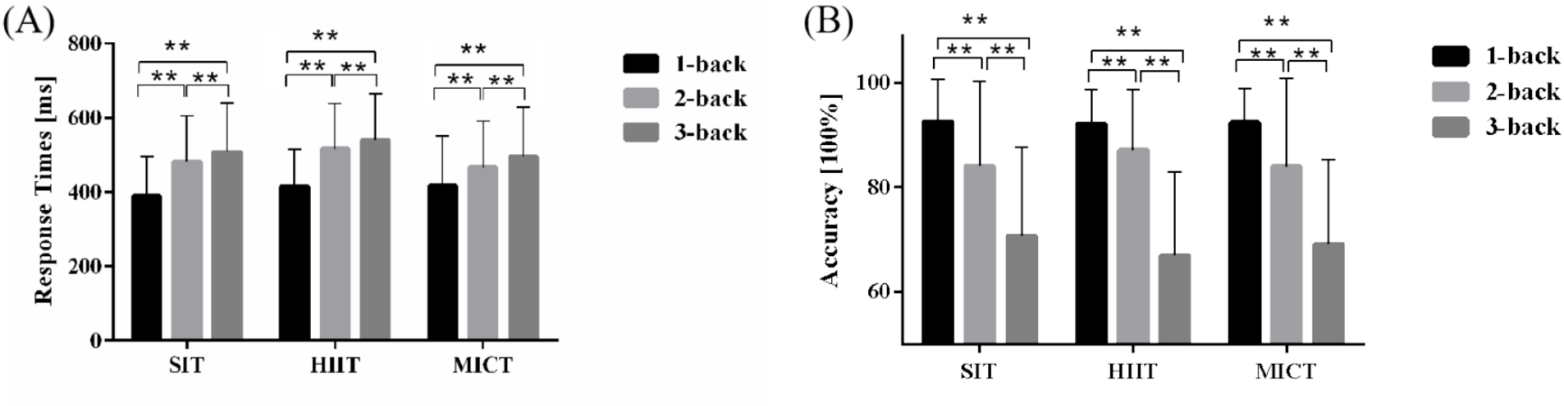
RT and ACC of the n-back task. **(A)** Comparison of RT among SIT, HIIT and MICT. **(B)** Comparison of accuracy among SIT, HIIT and MICT. Data are expressed as mean ± standard error. * indicates *p* < 0.05; ** indicates *p* < 0.001.

For ACC, as illustrated in Figure 3B, the RM-ANOVA revealed that the main effects of the task were significant [*F*_(2, 96)_ = 123.46, *p* < 0.001, *η^2^* = 0.72]. *Post hoc* tests showed that the 1-back task (92.36 ± 7.03%) was significantly higher than the 2-back task (85.03 ± 14.99%), t (146) = −9.57, *p* < 0.001, and 3-back task (68.95 ± 16.44%), t (146) = −9.65, *p* < 0.001. The ACC of the 2-back task was significantly higher than that of the 3-back task, t (146) = −2.70, *p* < 0.001. No main effects were observed involving experimental conditions [*F*_(2, 96)_ = 0.07, *p* = 0.93, *η^2^* = 0.001], and no interaction was observed involving experimental conditions and task [*F*_(4, 192)_ = 2.32, *p* = 0.06, *η^2^* = 0.046].

For RT, as depicted in Figure 3A, the RM-ANOVA revealed that the main effects of the task were significant [*F*_(2, 96)_ = 42.03, *p* < 0.001, *η^2^* = 0.47]. The RT of the 1-back task (410.58 ± 114.66 ms) was significantly faster than that of the 2-back task (488.35 ± 124.51 ms), t (146) = 6.54, *p* < 0.001, and 3-back task (514.79 ± 130.66 ms), t (146) = 19.26, *p* < 0.001. The RT of the 2-back task was significantly faster than that of the 3-back task, t (146) = 13.07, *p* < 0.001. The analysis revealed main effects of experimental condition [F _(2, 96)_ = 3.34, *p* = 0.04, *η^2^* = 0.07]. *Post hoc* tests showed longer RT for HIIT (491.16 ± 113.67 ms) relative to MICT (459.86 ± 106.08 ms), t (96) = 2.02, *p* = 0.046. No such difference was observed between SIT (462.71 ± 121.34 ms) and MICT. No interaction was observed involving experimental conditions and task [*F*_(4, 192)_ = 1.96, *p* = 0.10, *η^2^* = 0.039].

### 3.3 FS and FAS results

**Figure 4:**
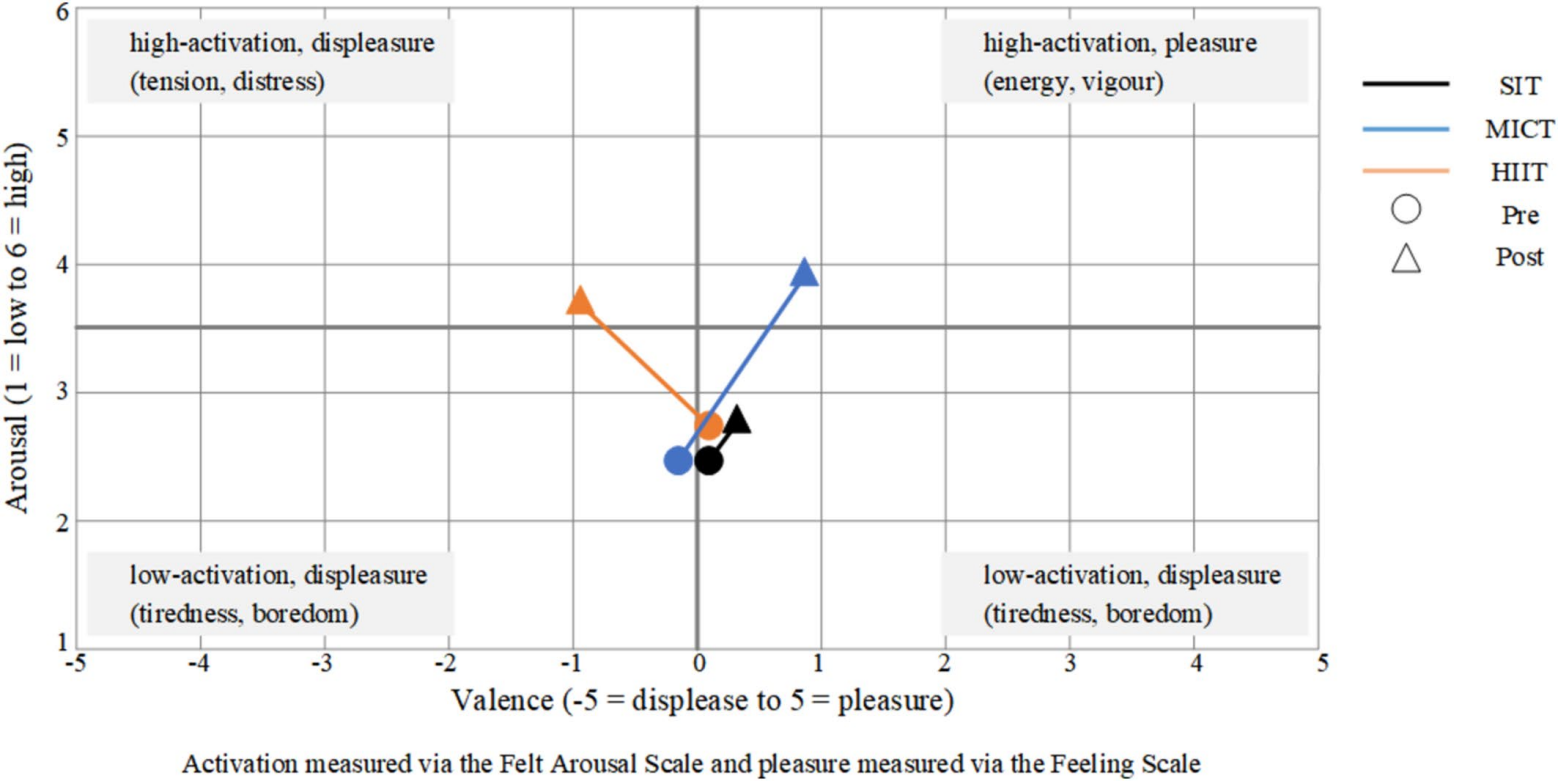
Affective response during the two conditions plotted in a three-dimensional space. Horizontal axis represents affective valence (FS) and the vertical axis represents perceived activation (FAS).

The circumplex model (Figure 4) depicts the variations in FAS and FS impacts before and after different intensity groups.

FAS analyses, conducted on the group’s FAS, revealed a significant main interaction effect *F*_(2, 96)_ = 17.55, *p* < 0.001, *η^2^* = 0.27, and main effects for condition (pre-post) *F* (1, 48) = 68.77, *p* < 0.001, *η^2^* = 0.59, and group *F*_(2, 96)_ = 7.36, *p* = 0.001, *η^2^* = 0.14. *Post hoc* analysis showed that post led to a significant increase in FAS scores (HIIT: 2.39 ± 1.30 to 3.76 ± 1.12, *p* < 0.001; MICT: 2.55 ± 0.88 to 3.94 ± 1.08, *p* < 0.001) compared to pre. Furthermore, HIIT resulted in significantly higher increases in FAS scores than SIT (*p* = 0.011), and MICT resulted in significantly higher increases in FAS scores than SIT (*p* < 0.01).

FS analyses, conducted on the group’s FS, revealed a significant main interaction effect F (2, 96) = 18.55, *p* < 0.001, *η^2^* = 0.28, and group F _(2, 96)_ = 5.77, *p* = 0.004, *η^2^* = 0.11. Post hoc analysis showed that post led to a significant increase in FS scores (SIT: 0.32 ± 1.38 to 0.58 ± 1.53, *p* = 0.01; HIIT: 0.18 ± 1.33 to −0.67 ± 1.85, *p* = 0.02; MICT: −0.27 ± 1.13 to 1.06 ± 1.79, *p* < 0.001) compared to pre. Furthermore, HIIT resulted in significantly higher decreases in FS scores than SIT (*p* = 0.01), and MICT resulted in significantly higher increases in FS scores than HIIT (*p* = 0.002).

## 4 Discussion

The present study investigated the acute effects of HIIT, MICT, and SIT on working memory and emotional states in adolescent females with subthreshold depression. The findings demonstrated that MICT had the most consistent positive effects, significantly improving reaction time in working memory tasks and enhancing emotional states, particularly self-reported pleasure and arousal. In contrast, HIIT elicited mixed emotional responses and showed limited cognitive benefits, while SIT had minimal impact on both domains. These results highlight the potential of moderate-intensity exercise as a practical and effective intervention for this population.

### 4.1 Effects on working memory performance

Research has consistently indicated that acute exercise interventions, such as MICT and HIIT, can influence reaction speed in working memory tasks (45, 46). In the present study, the RT of subjects following HIIT was significantly longer than that observed after SIT, whereas MICT showed no significant difference compared to SIT. These studies indicate that MICT and HIIT do not always consistently improve RT in working memory tasks. However, in terms of ACC, this study found no significant differences among the three intervention methods. Although some studies report conflicting results (47), meta-analytic evidence has suggested that acute MICT exerts low to moderate adverse effects on ACC in working memory tasks (48), underscoring the variability in individual responses to such interventions.

The neural mechanisms underlying these effects have been explored in prior studies. For example, acute MICT has been shown to enhance activation in brain regions such as the upper left parietal lobe and the lower right parietal lobe, which are critical for working memory (49, 50). However, these neural activations do not always translate into improved behavioral performance (51). This aligns with findings from this study, where MICT slightly shortened RT compared to SIT but failed to achieve statistical significance. It suggests that while a single session of MICT may activate brain regions associated with working memory, the degree of activation may not be sufficient to affect task performance significantly.

For HIIT, prior research has highlighted its potential to benefit executive function through efficient neural activation during shorter interventions (45). However, in this study, the 20-min HIIT session may have exceeded the optimal intensity-duration threshold, as evidenced by the inverted U-shaped relationship between exercise intensity, duration, and executive function (52–54). This could account for the decline in working memory performance observed post-HIIT, potentially attributed to exercise-induced fatigue (55). Moreover, similarities in brain impairments between individuals with subthreshold depression and those with clinical depression may further limit the working memory benefits observed in this population (9, 56).

### 4.2 Effects on emotional effects

The emotional outcomes of the interventions revealed notable differences among the exercise conditions. MICT elicited the most consistent improvements, significantly enhancing self-reported pleasure and moderately increasing arousal. These results are consistent with existing literature, which identifies moderate-intensity exercise as optimal for emotional benefits due to its balanced physiological demands (57). Conversely, HIIT produced mixed emotional responses. In this study, exercise intensity may be an important factor in regulating the subjects’ sense of pleasure, and the emotional benefits of exercise may be affected by intensity (58), particularly for vulnerable populations such as adolescents with subthreshold depression. Studies have found that keeping HIIT intensity at 80% or about 80% of HRmax may alleviate the negative emotions brought by HIIT (59). In contrast, SIT had minimal impact on both cognitive and emotional outcomes, which was anticipated. This finding reinforces the necessity of active exercise to stimulate psychological and cognitive benefits, highlighting the limited effectiveness of sedentary activities in addressing the needs of this population.

### 4.3 Limitation

While this study contributes to understanding the acute effects of exercise on working memory and emotional states in adolescent females with subthreshold depression, certain limitations should be acknowledged. Firstly, the homogeneous sample consisting only of adolescent females may limit the generalizability of the findings. Future research could benefit from including a more diverse sample to explore potential sex-based and developmental differences. Secondly, the cross-sectional design precludes conclusions about the long-term effects of exercise interventions. Longitudinal studies are recommended to assess whether the observed acute effects persist over time. Lastly, this study did not account for certain variables, such as baseline physical fitness levels or external factors like academic stress and sleep patterns, which might have influenced the results. Controlling these variables in future research would enhance the robustness and applicability of findings. By addressing these limitations, future studies can build upon the current findings to further refine exercise-based interventions and maximize their effectiveness in improving emotional and cognitive outcomes in adolescents with subthreshold depression.

## 5 Conclusion

In conclusion, this study suggests that acute MICT positively influences pleasure in adolescent women with subthreshold depression, while HIIT may have a negative impact. Future research should investigate maintaining HIIT intensity at 80% HR_max_ to potentially mitigate negative emotions. The study underscores the need for continuous exercise interventions to track working memory and emotional states in adolescent women with subthreshold depression.

## Data Availability

The datasets presented in this study can be found in online repositories. The names of the repository/repositories and accession number(s) can be found in the article/supplementary material.
